# Effect of Intermittent versus Chronic Calorie Restriction on Tumor Incidence: A Systematic Review and Meta-Analysis of Animal Studies

**DOI:** 10.1038/srep33739

**Published:** 2016-09-22

**Authors:** Yalan Chen, Lifeng Ling, Guanglei Su, Ming Han, Xikang Fan, Pengcheng Xun, Guangfei Xu

**Affiliations:** 1 Department of Nutrition and Food Science, School of Public Health, Nantong University, Nantong, Jiangsu, China; 2 Department of Medical Informatics, School of Medicine, Nantong University, Nantong, Jiangsu, China; 3 Department of Human Resources, Nantong University, Nantong, Jiangsu, China; 4 Department of Epidemiology and Biostatistics, School of Public Health-Bloomington, Indiana University, Bloomington, IN, USA

## Abstract

Both chronic calorie restriction (CCR) and intermittent calorie restriction (ICR) have shown anticancer effects. However, the direct evidence comparing ICR to CCR with respect to cancer prevention is controversial and inconclusive. PubMed and Web of Science were searched on November 25, 2015. The relative risk (RR) [95% confidence interval (CI)] was calculated for tumor incidence, and the standardised mean difference (95% CI) was computed for levels of serum insulin-like growth factor-1 (IGF-1), leptin, and adiponectin using a random-effects meta-analysis. Sixteen studies were identified, including 11 using genetically engineered mouse models (908 animals with 38–76 weeks of follow-up) and 5 using chemically induced rat models (379 animals with 7–18 weeks of follow-up). Compared to CCR, ICR decreased tumor incidence in genetically engineered models (RR = 0.57; 95% CI: 0.37, 0.88) but increased the risk in chemically induced models (RR = 1.53, 95% CI: 1.13, 2.06). It appears that ICR decreases IGF-1 and leptin and increases adiponectin in genetically engineered models. Thus, the evidence suggests that ICR exerts greater anticancer effect in genetically engineered mouse models but weaker cancer prevention benefit in chemically induced rat models as compared to CCR. Further studies are warranted to confirm our findings and elucidate the mechanisms responsible for these effects.

Cancer is, to some extent, a preventable disease that is presumably caused by a combination of genetic, environmental, and behavioural factors^1^. Several reviews have discussed how diet and nutrition contribute to human cancer risk^2^,^3^,^4^,^5^,^6^ by affecting the initiation, promotion and progression of cancers^7^,^8^,^9^. Two main types of dietary restriction are chronic calorie restriction (CCR) and intermittent calorie restriction (ICR) (e.g., intermittent fasting, alternate-day fasting, or routine periodic fasting)^10^,^11^,^12^,^13^. Two population-based studies have found a linear and inverse association between CCR and breast cancer risk^14^,^15^. However, because CCR requires constant food restriction, the tolerance and compliance for fasting is unsatisfactory; therefore, the effect of CCR might not be as good as expected. Researchers have been looking for more feasible styles of calorie restriction (CR) with comparable or even superior results. Currently, ICR regimens have been found to be equivalent to, if not better than, CCR for weight loss, providing an alternative approach for weight loss that might be better suited to some individuals^16^. Several population studies have shown that ICR can improve indicators of chronic diseases (e.g., insulin sensitivity, high density lipoprotein cholesterol and fat oxidation)^17^,^18^,^19^. The question of whether ICR show better tumor inhibitory effects than CCR remains unanswered. Unfortunately, most research focuses on animal models. There is little evidence from human studies.

An animal study using Sprague-Dawley rats reported that a reduction of only 12% [88% of *ad libitum* (AL) intake] in total energy intake abolished the mammary carcinogenic differences between a high-fat and a low-fat diet that were observed in the AL intake groups^20^. Other animal studies using either rats or mice have documented up to a 95% decrease in mammary tumor incidence resulting from a 20–40% reduction in total energy intake^21^,^22^,^23^,^24^.

A meta-analysis recently published by Lv *et al*.^25^ discussed the cancer preventive efficacy of different dietary restriction strategies in all animal models and found that CCR was effective against cancer in animal experiments compared to AL feeding; 5 of the 8 related studies (62.5%) also revealed a positive anticancer effect for ICR, although the pooling was restricted because of data insufficiency. However, evidence regarding whether ICR can provide additional cancer protective effects as compared to CCR is limited and controversial. In genetically engineered animal models, several studies have found that ICR prevented cancer development to a greater extent than CCR^22^,^23^,^26^,^27^, while other studies did not^28^,^29^,^30^,^31^,^32^,^33^,^34^. In chemically induced models, several studies have indicated that ICR is less effective for cancer prevention than CCR^35^,^36^,^37^,^38^,^39^, and this difference was statistically significant in several studies^35^,^37^,^39^.

With the exception of this review, no meta-analysis or systematic review has been published that specifically compares the effects of ICR and CCR on cancer prevention according to the animal models used. Therefore, we quantitatively summarize the up-to-date literature to compare the cancer preventive effects of ICR and CCR in rodent models and explore the potential effect modification according to the animal model.

## Methods

### Literature search

A systematic review of the literature was performed by searching Pubmed and Web of Science for animal intervention studies published in English-language, peer-reviewed journals on November 25, 2015. The search terms included “intermittent fasting,” “alternate-day fasting,” “intermittent calorie restriction”, “weight cycle,” “cyclic food restriction”, or “intermittent energy restriction”, as well as “tumor incidence”, “tumorigenesis”, “cancerous”, “tumors”, “IGF-1” or “anti-cancer”. In addition, we reviewed studies in the reference lists of the retrieved studies and review articles to search for other potentially eligible studies.

### Inclusion and exclusion criteria

The inclusion criteria comprised the following: 1) the study was an intervention study; 2) the study used mice or rats as subjects; 3) the sample size in each group was at least 8; 4) the trial duration was at least 2 weeks; 5) the study examined the anticancer effect of ICR (intermittent fasting/alternate-day fasting) *vs*. CCR; 6) the study with any effect size for which 95% confidence intervals (CIs) were provided or such information could be derived; and 7) the primary endpoints of the study were tumorigenesis rate or number of tumors, tumor weight, age at detection, insulin-like growth factor-1 (IGF-1), leptin or adiponectin.

The exclusion criteria included the following requirements: 1) the study was an *in vitro* or a human study; 2) the study was an animal study but the subject was neither mice nor rats; 3) the study was not an intervention study; 4) the study combined CCR/ICR with other factors such as exercise, nutrition supplements, radiation or pharmaceuticals, *etc*.; 5) the trial duration of the study was less than 2 weeks; or 6) the study did not report any effect size with its 95% CI or such information could not be derived.

### Study selection and data extraction

The titles and abstracts of the obtained studies were first reviewed independently by two investigators (Y.C. and L.L.) to determine whether they met all of the inclusion criteria. Then, the full texts of the potentially included studies were investigated independently with reference to the inclusion and exclusion criteria. Two reviewers (Y.C. and L.L.) independently appraised each included article according to the Systematic Review Centre for Laboratory Animal Experimentation’s RoB tool, which is based on the Cochrane RoB tool and specifically designed for animal intervention studies^40^. This tool contains 10 entries (Supplementary Table 1) related to the following 6 types of bias: selection bias, performance bias, detection bias, attrition bias, reporting bias and other biases. Higher quality scores represent a lower risk of bias. Scores of 0–3, 4–7 and 8–10 represent high, moderate, and low risks, respectively. This study followed the Preferred Reporting Items for Systematic Reviews and Meta-Analyses (PRISMA) guidelines. The checklist can be found in Supplementary Table 2.

Data from the included studies were extracted by the two investigators (Y.C. and L.L.) using standardised and piloted design formats. Discrepancies in the process of study selection and data extraction were resolved through a group discussion with two other authors (P.X. and G.X.).

The data were independently examined and adjudicated after being extracted and assessed; several values were extracted from the results of original graphs in studies in which data were not provided directly in the text or tables using GetData Graph Digitizer^41^,^42^. The major outcomes and conclusions were extracted from each study using preset data recording forms. Baseline characteristics of the included studies are given in Supplementary Table 3. The information includes animal type, tumor type, feeding regimen, trial length and body weight at the end of the follow up. Tumor characteristics, including the number of subjects with tumors, age at detection, number of tumors per animal and tumor weight in each model and are shown in Tables 1 and 2. In addition, three tumor-related factors, including the hormones IGF-1, leptin, and adiponectin, were extracted.

### Statistical analysis

Tumor incidence was compared between ICR and CCR group in genetically engineered and chemically induced models, respectively. Based on the information extracted from each included study, the relative risk (RR) of developing a primary tumor was calculated as *p*_1_/*p*_0_, and the corresponding 95% CI was calculated as^43^:


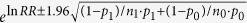


where *p*_1_ and *p*_0_ are tumor incidences in ICR and CCR group, respectively; and *n*_1_ and *n*_0_ are the related sample size in each group.

Then the RRs (95% CIs) were transformed into their natural logarithms to stabilize the variances and normalize their distributions. The pooled RRs with theirs 95% CIs were calculated by a random-effects model weighting for the inverse of the variance^44^.

The heterogeneity among studies was tested by Cochran’s *Q* test and quantified by the *I*^2^ statistic. An *I*^2^ value of <25%, 25–<50%, 50–<75% and ≥75% represents very low, low, moderate, and high heterogeneity, respectively^45^. A *P*-value of ≤0.10 was considered statistically significant. Subgroup or meta-regression analyses were used to identify possible sources of heterogeneity.

Publication bias was assessed by Egger’s regression asymmetry test (when the numbers of studies was ≥3) or Begg’s asymmetry test (when the numbers of studies was <3) with a significance level of 0.10. The Duval and Tweedie nonparametric “trim and fill” method was used to adjust for publication bias if needed^46^.

In addition, the standardised mean differences (SMDs) and 95% CIs were computed to compare the differences between the two groups (ICR *vs*. CCR) in the levels of serum IGF-1, leptin, and adiponectin that may be involved in the development of the tumor. Other major characteristics including tumor weight, number of tumors/animal, and age at detection, were compared using a similar approach between two groups.

In the sensitivity analyses, the influence of each included study on the pooling was examined by omitting one study at a time, and a random-effects model was replaced with a fixed-effects model to evaluate whether the model selection substantially affected the pooled results.

All analyses were performed using STATA (Version 14.0; STATA Corporation LP, College Station, Texas, USA). A two-sided *P* value of ≤0.05 was considered statistically significant, if not otherwise specified.

## Results

### Eligible studies

**Figure 1:**
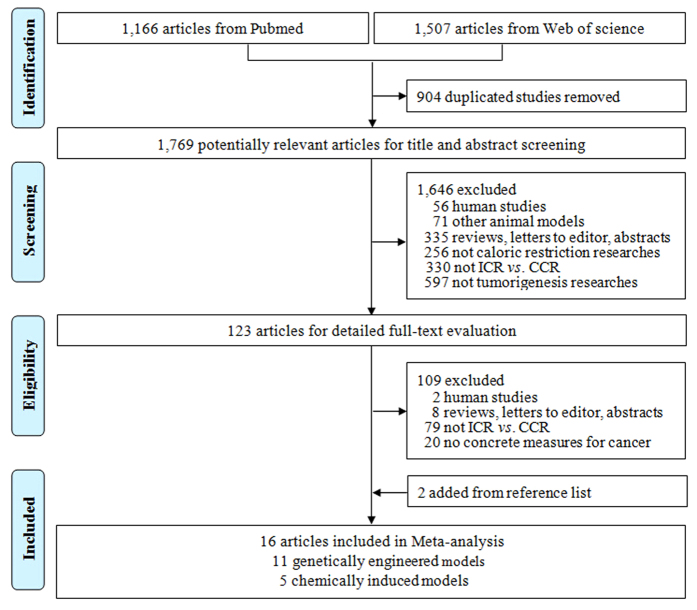
The selection process (PRISMA). CCR: chronic calorie restriction; ICR: intermittent calorie restriction.

The flow of the search strategy followed PRISMA and is shown in Fig. 1. A total of 2,673 studies were identified, 1,166 from Pubmed and 1,507 from Web of Science. In addition to the 904 duplicated studies, 1,646 studies were excluded after reviewing the title and abstract, and the details are documented in Fig. 1. Moreover, a total of 109 studies were excluded after full-text reading according to the inclusion criteria for one of the following reasons: human studies (*n* = 2), reviews, letters to editor, abstracts (*n* = 8), not a direct comparison of ICR with CCR (*n* = 79), or no concrete measures for cancer (*n* = 20). Furthermore, two studies were found from the relevant reference lists. Thus, a total of 16 animal studies were included in this meta-analysis. The score of quality assessment of the 16 studies using the Systematic Review Centre for Laboratory Animal Experimentation’s RoB tool ranged from 5 to 7 and all represent moderate quality and risks except for an unpublished study.

### Characteristics of studies

Of the 11 eligible studies included for genetically engineered models^22^,^23^,^26^,^27^,^28^,^29^,^30^,^31^,^32^,^33^,^34^ performed on mice (Table 1 and Supplementary Table 3), 8 (72.7%) focused on mammary cancer^22^,^23^,^26^,^27^,^30^,^31^,^33^,^34^, 1 on prostate cancer^29^, 1 on pancreatic cancer^32^ and 1 on multiple cancers^28^. The feeding regimens in the ICR groups were relatively homogenous, 10 of the 11 studies (90.9%)^22^,^23^,^26^,^27^,^29^,^30^,^31^,^32^,^33^,^34^ used the regimen “50% CR during the period of restriction (ICR-R) followed by an equal time of re-feeding at 100% AL intake (ICR-RF) with a time interval between 2 to 3 weeks. The energy intake in the CCR group ranged from 60% to 75% of the AL intake. A total of 7 (63.6%) studies^22^,^23^,^26^,^30^,^31^,^32^,^34^ used an AIN-93-modified diet, which had a 2-fold increase in protein, fat, vitamins, and minerals as compared to the original normal AIN-93M diet. The median trial length of all studies was 62 weeks with a range from 38^32^ to 76^22^ weeks.

Five studies were included that utilised chemically induced models^35^,^36^,^37^,^38^,^39^ performed on rats (Table 2). Three models used the carcinogen 7,12-dimethyIbenz-[fl]anthracene (DMBA)^35^,^36^,^37^ and two injected 1-methyl-1-nitrosourea (MNU)^38^,^39^. There was only 1 study that discussed multiple cancers^39^; all of the other studies focused on mammary cancer^35^,^36^,^37^,^38^ (Supplementary Table 3). Two^35^,^37^ of the 5 studies performed repeated cycles of 2 days of CR (60% AL intake) followed by 2 days of 100% AL feeding in the ICR group; one study^38^ used cycles of 1 week with 67% AL intake followed by 3 weeks with 100% AL intake; one study^39^ used 6 weeks with 60% AL intake and 8 days of 100% AL intake; and the remaining study^36^ used 1.75 months with 25% AL intake and 2.25 months with 100% AL intake. All CCR groups were based on 60% AL intake except one with 75% AL intake^36^. The time of the experimental stage ranged from 7^39^ to 18^38^ weeks, a relatively shorter time than that in the genetically engineered models.

### Effect of ICR versus CCR on tumor incidence

**Figure 2:**
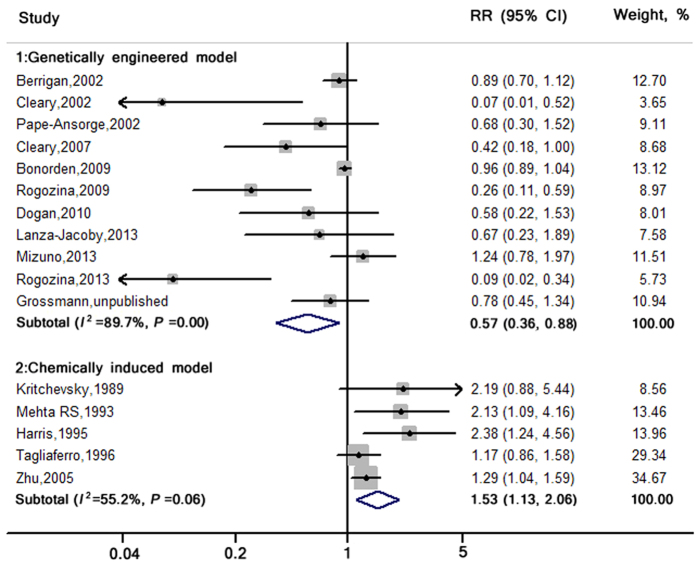
RRs and 95% CIs for tumor incidence in genetically engineered and chemically induced animal models. The pooled estimates were obtained using a random-effects model. The dots indicate the RRs comparing ICR to CCR. The size of the shaded square is proportional to the weight in each study. The horizontal lines represent the 95% CIs. The diamond data markers indicate the pooled RRs with corresponding 95% CIs. CCR: chronic calorie restriction; CI: confidence interval; ICR: intermittent calorie restriction; RR: relative risk (for tumor incidence).

The tumor incidence results in two models are demonstrated by a forest plot (Fig. 2). Eleven^22^,^23^,^26^,^27^,^28^,^29^,^30^,^31^,^32^,^33^,^34^ of 16 studies used genetically engineered models, and the remaining five used chemically induced models^35^,^36^,^37^,^38^,^39^.

As presented in Tables 1 and 2 and Fig. 2, a total of 908 mice (469 in the ICR group and 439 in the CCR group) with 374 events (161 in the ICR group and 213 in the CCR group) were included in the genetically engineered models. Comparing ICR to CCR, the pooled RR (95% CIs) of tumor incidence was 0.57 (0.37, 0.88) with high heterogeneity (*I*^2^ = 89.7%, *P *< 0.01). Because strong evidence of publication bias was observed (Egger’s test: *P *< 0.01), the adjusted pooled association was 0.66 (0.50, 0.88) using the Duval and Tweedie method.

In chemically induced models, a total of 379 rats (216 in the ICR group and 163 in the CCR group) were included, and the total events were 128 and 73 for the ICR and CCR group, respectively. The pooled RR (95% CIs) of tumor incidence comparing ICR to CCR was 1.53 (1.13, 2.06), with moderate heterogeneity (*I*^2^ = 55.2%, *P* = 0.06). Because publication bias was documented (Egger’s test: *P* = 0.052), the pooled association was adjusted as 1.33 (1.02, 1.74) using the Duval and Tweedie method.

### Effect of ICR versus CCR on levels of IGF-1, leptin, and adiponectin

**Figure 3:**
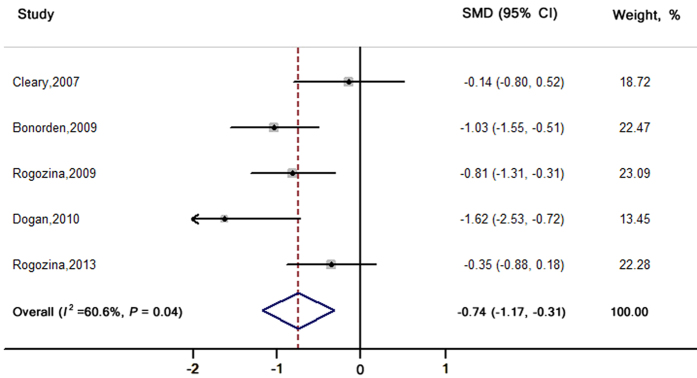
SMDs and 95% CIs for serum IGF-1 levels in genetically engineered animal models. The pooled estimates were obtained using a random-effects model. The dots indicate the SMDs comparing ICR-R to CCR. The size of the shaded square is proportional to the weight of each study. The horizontal lines represent the 95% CIs. The diamond data markers indicate the pooled SMDs with corresponding 95% CIs. CCR: chronic calorie restriction; CI: confidence interval; ICR: intermittent calorie restriction; ICR-R: restriction period in ICR group; SMD: standardised mean difference.

Five^22^,^26^,^27^,^29^,^31^ out of the 11 studies (Fig. 3) that used genetically engineered models compared levels of IGF-1 between ICR-R and CCR. All of them focused on 2 types of hormone-sensitive cancers - mammary and prostate cancer. The pooled SMD was −0.74 (−1.17, −0.31) with moderate heterogeneity (*I*^2^ = 60.6%; *P* = 0.04). No publication bias was found (Egger’s test: *P* = 0.60).

**Figure 4:**
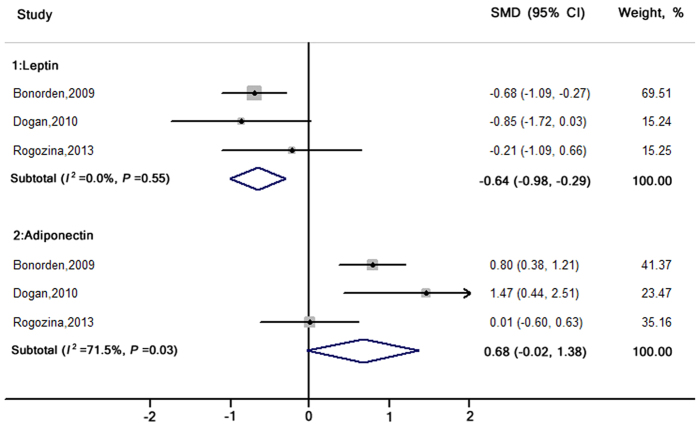
SMDs and 95% CIs for serum leptin and adiponectin levels in genetically engineered animal models. The pooled estimates were obtained using a random-effects model. The dots indicate the SMDs comparing ICR-R to CCR. The size of the shaded square is proportional to the weight of each study. The horizontal lines represent the 95% CIs. The diamond data markers indicate the pooled SMDs with corresponding 95% CIs. CCR: chronic calorie restriction; CI, confidence interval; ICR: intermittent calorie restriction; ICR-R: restriction period in ICR group; SMD: standardised mean difference.

Three studies^27^,^29^,^31^ (Fig. 4) reported levels of leptin and adiponectin and the pooled SMD was −0.64 (−0.98, −0.29) and −0.68 (−0.02, 1.38), respectively. No heterogeneity was found for pooling leptin (*I*^2^ = 0.0%, *P* = 0.55), but moderate heterogeneity was documented in pooling adiponectin (*I*^2^ = 71.5%, *P* = 0.03). No evidence of publication bias was found for pooling leptin and adiponectin (Egger’s test: *P* = 0.75 and *P* = 0.88).

Only 1 study that used a chemically induced model had data available on IGF-1 levels, and none of the studies reported data on levels of leptin, and adiponectin, which limited our ability to pool them quantitatively.

### Effect of ICR versus CCR on other tumor characteristics

All of the other pooled statistical effects of tumor characteristics and tumor relative indexes are displayed in Table 3.

In the studies utilizing genetically engineered models, three studies have complete data descriptions (mean ± SE/SD) on tumor weight^22^,^26^,^34^, number of tumors/animal^22^,^27^,^34^, and age at detection^22^,^26^,^34^. No significant difference was found in tumors per animal between the 2 groups with pooled SMD (95% CIs) [−0.51 (−1.14, 0.12); *I*^2^ = 0.0%; *P* = 0.65], tumor weight [0.15 (−0.65, 0.94); *I*^2^ = 50.2%; *P* = 0.13] or age at detection [0.17 (−0.38, 0.72); *I*^2^ = 0.0%; *P* = 0.40]. No publication bias was found for tumors per animal and tumor weight pooling (Egger’s test: *P* = 0.57; *P* = 0.89) but was found for the age at tumor detection (Egger’s test: *P* = 0.05).

In the chemically induced models, two studies reported information on the number of tumors/animal^38^,^39^. No significant difference was found [0.63 (−1.31, 2.57)], and publication bias (Begg’s test: *P* = 1.00) was not evident. However, high heterogeneity was observed (*I*^2^ = 95.2%; *P *< 0.01).

### Sensitivity analysis

The findings were generally consistent when using a fixed-effects model (Supplementary Table 4). Omitting 1 study each time and recalculating the pooled RRs/SMDs for the rest of the studies showed that none of the single studies substantially influenced the pooled RR for tumor incidence or the pooled SMDs for the other continuous outcomes (Supplementary Table 5).

## Discussion

The main findings of this study indicate that ICR showed a greater anticancer effect in genetically engineered mouse models but a weaker cancer prevention benefit in chemically induced rat models as compared to CCR. The decreased IGF-1 and leptin and increased adiponectin in genetically engineered models supported our main findings.

Compared to AL intake, anti-tumor benefits from CCR and ICR have been reported for breast, colon, liver, skin, and lung tumors in rodent models^32^,^47^,^48^,^49^. CCR of 30% or greater energy reduction consistently reduces tumor incidence in spontaneous^50^, chemically induced^47^ and radiation-induced tumor models^51^. However, no general conclusion could be drawn regarding the tumor inhibition of ICR compared with CCR.

It is worth noting that our findings (i.e., a greater anticancer effect in genetically engineered models and a weaker benefit on cancer prevention in chemically induced models relative to CCR) are consistent with those suggested by Thompson *et al*.^52^. The decreased IGF-1 and leptin and the increased adiponectin levels in genetically engineered models reflect the significantly superior tumor inhibition of ICR compared to CCR. Opposite inhibition effects can be observed when comparing ICR to CCR in the 2 animal models, indicating that CR may have different mechanisms when different tumor models are applied.

Although the exact mechanisms of the anticancer effect of CCR are debatable, it is widely believed that CCR prevents tumorigenesis by decreasing metabolic rate^53^ and promoting protective mechanisms that allow DNA damage to be prevented^54^. Simone *et al*.^55^ suggests that the mechanism behind ICR is relatively simple: it postpones tumor growth by starving tumors from glucose for a short period of time. A modified diet of increased protein and fat and decreased carbohydrates in the ICR group (similar to ketogenic diets) may account for a large proportion of the effects^56^. However, in addition to the widely studied dysregulated glucose metabolism to fuel tumor cell growth, accumulating evidence suggests that utilisation of amino acids and lipids also contributes significantly to cancer cell metabolism^57^,^58^. Whether these factors play similar roles in tumor inhibition with different models or whether they produce different suppression effects remains unclear.

### Tumor suppression in genetically engineered models

For genetically engineered models, a review by Varady *et al*.^59^ in 2007 suggested a protective effect of ICR on cancer risk, which supports our findings. However, we found no statistically significant difference in the number of tumors per animal and tumor weight comparing ICR to CCR group, which suggests that ICR may play a protective role only in the early stage of tumorigenesis. We speculate that this limitation is partially due to the more severe late tumor development in the ICR group.

Research shows that the IGF-1 receptor and the insulin receptor may differ mechanistically in subjects undergoing ICR compared with those undergoing CCR and this may result in greater reductions in hepatic and visceral fat stores, IGF-1 levels, leptin and cell proliferation, and increase insulin sensitivity and adiponectin levels^26^,^31^,^60^.

A growing body of evidence suggests that insulin and IGF-1 receptors regulate cell proliferation, differentiation, apoptosis, glucose transport, and energy metabolism by regulating downstream signalling cascades through insulin receptor substrate molecules. CR in rodents reduces IGF-1/insulin–phosphatidylinositol-3 kinase-Akt-mammalian target of rapamycin complex 1 signalling, which has been shown to be correlated with significant tumor growth delay^61^,^62^.

Nevertheless, although CR generally reduces the levels of IGF-1 and leptin, their absolute values were higher in the ICR group than in the CCR group in most of our studies^23^,^28^,^32^,^34^, which indicates an inverse correlation between the levels of IGF-1 and leptin and tumor occurrence. From several studies that reported information stratified by period (ICR-R *vs*. ICR-RF)^22^,^26^,^27^,^29^,^31^, we were able to explain the above results. Although the levels of IGF-1 and leptin during the ICR-R period were much lower compared to the CCR group, they increased substantially during the ICR-RF period and became much higher than those in the CCR group. This could explain the higher mean levels of IGF-1 and leptin in the ICR group than those in the CCR group (Table 1).

The sharply reduced serum IGF-1 and leptin and the elevated adiponectin and adiponectin/leptin ratio (data not shown) were associated with the protective effects of ICR in genetically engineered models. Furthermore, previous studies have characterised IGF-1 and leptin as mediators of the anticancer effects of CR^63^.

Leptin is an activator of cell proliferation and anti-apoptosis in several cell types and an inducer of cancer stem cells; its critical roles in tumorigenesis are based on its oncogenic, mitogenic, proinflammatory, and proangiogenic actions^64^.

Leptin enhances proliferation of human cancer cell lines^65^,^66^. In contrast, adiponectin reduces cancer cell proliferation^67^,^68^, which is verified by the results of our study. Findings from two human studies^68^ and one *in vitro* study that evaluated the impact of different adiponectin/leptin ratios on human breast cancer cell proliferation^69^ suggested that the adiponectin/leptin ratio may be more important in determining how these two proteins together affect mammary tumor development than either one alone. Two studies of ICR-R *vs*. CCR found that ICR can promote a better adiponectin/leptin ratio than CCR (15.5 ± 5.6 *vs*. 3.8 ± 0.8^31^; 7.96 ± 2.6 *vs*. 2.85 ± 0.3^27^). The reduced serum leptin and elevated adiponectin/leptin ratio were associated with the protective effect of ICR^63^.

### Tumor suppression in chemically induced models

Chemically engineered models are induced by chemical carcinogens such as polycyclic aromatic hydrocarbon (PAH), N-nitrosamines and mycotoxin^70^. Most of the studies included in this meta-analysis used the carcinogen DMBA, a type of PAH known to cause mammary tumors in rats. The formation of PAH-DNA adducts (DNA binding products), a necessary step in PAH-initiated carcinogenesis, has been widely studied in experimental models and has been documented in human tissues^71^. According to previous studies, several nutrients such as vitamin A^72^,^73^, C^74^, D^75^,^76^ and E^77^,^78^ protect against the carcinogenic effects of DMBA exposure. The relatively low intake of these nutrients during the ICR-R period could cause a reduced protective effect, which may eventually lead to a relatively high tumor occurrence. More importantly, tumor cells have evolved the ability to utilize different carbon sources due to the limited supply of nutrients. For example, glutamine, the most abundant amino acid in the plasma, has long been recognized as an alternative fuel^57^.

### Advantages and disadvantages

There are several strengths in this research that should be highlighted. First, we collected and systematically analysed the most up-to-date comprehensive evidence and performed the first quantitative meta-analysis to compare the anticancer effects between ICR and CCR. Second, all of the included studies are intervention studies, which provide stronger evidence than observational studies. Third, to our knowledge, this is the first study conducted to identify animal models (genetically engineered models *vs*. chemically induced models) that study the anticancer effects of ICR versus those of CCR.

When interpreting our results, a number of issues should be considered. First, the frequency of cycling (the number of cycles experienced), the duration of the cycles and the restriction regimen (the amount of CR) varied across studies and need further unification. However, we used random-effects models in accordance with the heterogeneity, and further adjusting for these factors using meta-regression did not substantially change our main findings. Second, several studies divided the ICR group into the ICR-R period and the ICR-RF period, while others did not, which might cofound our findings. However, the likelihood of this is low, because the results were generally consistent when we restricted our analysis to the ICR-R period in the studies in which such information was available. Third, although we did not find strong evidence of publication bias in most of the pooling and we adjusted the pooled association using statistical methods when publication bias existed, publication bias due to unpublished data or publications in non-English languages may exist. Fourth, all genetically engineered studies used mice and all chemically induced models used rats, and mice may respond better to ICR than rats, which might partially explain our results or at least this possibility cannot be completely ruled out. Finally, this research focused on studies conducted in rodents, which limits the border application of the findings. Thus, the results need to be further verified in other advanced animals, e.g., mammals and primates, and in human beings. It is also important to note that the human studies examined in this review are not sufficient; the direct effect of ICR *vs*. CCR on cancer has been tested only in animal models. Future studies with more reasonable experimental designs are needed to answer these important questions. Much work remains to be done to translate the knowledge gained from CR research to humans for chronic disease prevention^79^.

### Highlights and implications

Based on the evidences herein, we propose that, for individuals carrying several of the cancer risk genes, ICR may be a more effective choice; for the chemical carcinogen-exposed population group, CCR may achieve better results. This finding also suggests that the energy control in the ICR-R period needs to be confined to a reasonable range and that supplementation of certain protective nutrients during the ICR-R period would achieve better suppression effects, which has been mentioned in one cancer chemoprevention study^80^ but requires further experimental and clinical verification. Second, it needs to be emphasized that in the presence of strong carcinogens, the excessive restriction of energy and total nutrition may lead to excessive loss of several nutrients that are beneficial to anti-cancer mechanisms and this may eventually dilute the anti-cancer effect of energy restriction. Third, original, randomized controlled trials are needed to directly compare the anticancer effect of a specific ICR regimen with that of a specific CCR regimen, considering specific tumor occurrence and development and using both genetically engineered and chemically induced models. The research on ICR needs further refinement. For instance, it is necessary to develop a clearer definition of the ICR-R and ICR-RF periods and indicators of the two periods. Studies can also be designed to compare the cancer inhibition effects of ICR and CCR using specific animal species and induced models. This will help us better answer several questions about the tumor inhibition of ICR. For example, can the acquired protective effect in the restriction period be compromised by AL intake or high fat intake in the re-feeding period? If the answer is yes, can the compromised effects be modified using different animal models (genetic *vs*. chemical models)? Furthermore, is the attenuation effect stronger in the re-feeding period in the chemically induced model than in the genetically engineered model?

Important questions remain unanswered. For example, would greater energy restriction in the ICR group attain equivalent or superior tumor inhibition in the DMBA-induced animal cancer models than CCR? This question requires further verification in both experimental and clinical studies and leads to further questions: Is it comprised by AL intake in the re-feeding period? What is the difference between ICR and CCR in inhibiting tumors when different fuel sources are used? Can ICR provide additional cancer protective effects in human tumors as compared to CCR, and what is the long-term safety of ICR? Answering these questions will provide helpful information for dietary recommendations for tumor prevention and for weight maintenance/control in normal weight/overweight individuals.

### Summary

The protective effects of ICR and CCR on tumor varied according to the animal model. Compared to CCR, ICR could prevent cancer to a greater extent in genetically engineered mouse models and to a lesser extent in chemically induced rat models. The potential difference in the mechanism of the effects of ICR *vs*. CCR in different tumor exposure scenarios, including genetic defects and environmental exposures, warrants further elucidation, which may facilitate the adoption of ICR for human beings.

## Additional Information

**How to cite this article**: Chen, Y. *et al*. Effect of Intermittent versus Chronic Calorie Restriction on Tumor Incidence: A Systematic Review and Meta-Analysis of Animal Studies. *Sci. Rep.* **6**, 33739; doi: 10.1038/srep33739 (2016).

## Supplementary Material

Supplementary Information

## Figures and Tables

**Table 1:** Characteristics of studies included in genetically engineered mouse models.

Source	Group	Tumor characteristics				IGF-1, ng/ml	Leptin, ng/ml	Adiponectin, μg/ml
		No. with tumor/subjects	Age at detection, week	No. of tumors/animal	Weight, g			
Berrigan^28^	ICR	24/31	NA	NA	NA	473 ± 54.1	14.7 ± 3.7	NA
	CCR	27/31	NA	NA	NA	412 ± 24.1	4.8 ± 1.7	NA
Cleary^23^	ICR	1/30	80	1.0	0.06	553.6 ± 37.5	NA	NA
	CCR	15/33	73.5 ± 2.2	1.7 ± 0.3	0.67 ± 0.13	467.7 ± 22.5	NA	NA
Pape-Ansorge^34^	ICR	7/31	56.9 ± 6.7	1.3 ± 0.2	1.29 ± 0.27	626 ± 16	NA	NA
	CCR	11/33	54.0 ± 4.1	1.7 ± 0.2	1.69 ± 0.16	567 ± 21	NA	NA
Cleary^22^	ICR	6/39	79.4 ± 0.3	1.6 ± 0.4	0.48 ± 0.25	R:369 ± 28 RF:457 ± 22	NA	NA
	CCR	11/30	74.5 ± 2.6	1.8 ± 0.4	0.24 ± 0.09	383 ± 20	NA	NA
Bonorden^29^	ICR	92/101	38	NA	NA	R:296.9 ± 18.4* RF:322.7 ± 14.8*	R:1.2 ± 0.2* RF:2.8 ± 0.3*	R:18.3 ± 1.9* RF:16.2 ± 1.7*
	CCR	75/79	35	NA	NA	388.8 ± 12.2*	2.6 ± 0.3*	11.4 ± 0.8*
Rogozina^26^	ICR	6/66	67.0 ± 4.2	1.0	0.66 ± 0.38	R:476.8 ± 38.9* RF:656.8 ± 29.2*	NA	NA
	CCR	23/65	69.0 ± 1.6	1.39 ± 0.2	0.43 ± 0.04	630.9 ± 27.8*	NA	NA
Dogan^31^	ICR	6/52	55	NA	NA	R:195 ± 6 RF:211 ± 21	R:2.6 ± 0.4 RF:2.9 ± 0.7	R:25.1 ± 3.5 RF:17.5 ± 2.2
	CCR	8/40	37	NA	NA	298 ± 21	5.4 ± 1.1	14.7 ± 1.2
Lanza-Jacoby^32^	ICR	4/15	NA	NA	NA	284.1 ± 17.9	1.2 ± 0.2	9.9 ± 0.6
	CCR	6/15	NA	NA	NA	226.4 ± 12.2	0.9 ± 0.2	9.7 ± 0.6
Mizuno^33^	ICR	17/29	49.1	NA	1.4	386.5 ± 73.6*	0.8 ± 0.3*	7.4 ± 0.2*
	CCR	17/36	52.4	NA	1.5	656.4 ± 49.1*	3 ± 0.6*	6.2 ± 0.5*
Rogozina^27^	ICR	2/45	82.0 ± 0.0	1.0 ± 0.001	0.05 ± 0.0	R:231.4 ± 66.0* RF:382.6 ± 22.7*	R:3.2 ± 2.1* RF:13.2 ± 8.7*	R:11.5 ± 4.4* RF:8.1 ± 4.3*
	CCR	23/44	74.5 ± 1.6	1.7 ± 0.16	0.75 ± 0.24	305.2 ± 19.6*	11.0 ± 10.1*	11.2 ± 5.9*
Grossmann^30^	ICR	12/30	NA	NA	NA	NA	NA	NA
	CCR	17/33	NA	NA	NA	NA	NA	NA

Values are means ± SE.

CCR: chronic calorie restriction; ICR: intermittent calorie restriction; IGF-1: insulin-like growth factor 1; NA: not available; R: results of ICR restriction periods; RF: results of ICR refeeding periods; SE: standard error.

^*^Data were extracted from the original figures using GetData Graph Digitizer.

**Table 2:** Characteristics of studies included in chemically induced rat models.

Source	Group	Inducer	Tumor characteristics			IGF-1, ng/ml
			No. with tumors/subjects	No. of tumors/animal	Weight, g	
Kritchevsky^36^	ICR	DMBA	35/80	NA	NA	NA
	CCR	DMBA	4/20	NA	NA	NA
Mehta^37^	ICR	DMBA	17/30	3.47	NA	NA
	CCR	DMBA	8/30	2.75	NA	NA
Harris^35^	ICR	DMBA	19/30	2.11	NA	NA
	CCR	DMBA	8/30	1.75	NA	NA
Tagliaferro^38^	ICR	Nmethyl-n-nitrosourea	37/56	2.3 ± 0.4	12.9 ± 3.0	NA
	CCR	Nmethyl-n-nitrosourea	30/53	3.1 ± 0.4	14.2 ± 3.0	NA
Zhu^39^	ICR	1-methyl-1-nitrosourea	20/20	4.7 ± 0.6	NA	367.1 ± 31.8*
	CCR	1-methyl-1-nitrosourea	23/30	1.5 ± 0.2	NA	172.9 ± 14.1*

Values are means ± SE.

DMBA: 7,12-dimethylbenz[a]anthracene; IGF-1: insulin-like growth factor 1; NA: not available; SE: standard error.

^*^Data were extracted from the original figures using GetData Graph Digitizer.

**Table 3:** Standardised mean differences in other tumor relative indexes comparing ICR to CCR in two animal models.

Indexes	No. of studies	No. of subjects (ICR/CCR)	SMD (95% CI)	Heterogeneity test
**Genetically engineered mouse model**				
Age at detection	3	19/45	0.17 (−0.38, 0.72)	*P* = 0.40, *I*^*2*^ = 0.0%
No. of tumors/animal	3	15/45	−0.51 (−1.14, 0.12)	*P* = 0.65, *I*^*2*^ = 0.0%
Tumor weight	3	19/45	0.15 (−0.65, 0.94)	*P* = 0.13, *I*^*2*^ = 50.2%
**Chemically induced rat model**				
No. of tumors/animal	2	57/53	0.63 (−1.31, 2.57)	*P* < 0.00, *I*^*2*^ = 95.2%

All the pooled estimates were obtained using a random-effects model.

CCR: chronic calorie restriction; CI: confidence interval; ICR: intermittent calorie restriction; SMD: standardised mean difference.

## References

[b1] WCRF/AICR. Food, nutrition, physical activity and the prevention of cancer:a global perspective. http://wcrf.org/sites/default/files/Second-Expert-Report.pdf, (2007) (Date of access: 18/04/2016).

[b2] Goldman R. & Shields P. G. Food mutagens. J Nutr. 133 Suppl 3, 965s–973s (2003). 12612183 10.1093/jn/133.3.965S

[b3] Aragon F., Perdigon G. & de Moreno de LeBlanc A. Modification in the diet can induce beneficial effects against breast cancer. World J Clin Oncol. 5, 455–464 (2014). 25114859 10.5306/wjco.v5.i3.455 PMC4127615

[b4] Bazzan A. J., Newberg A. B., Cho W. C. & Monti D. A. Diet and nutrition in cancer survivorship and palliative care. Evid Based Complement Alternat Med: CAM 2013, e917647, 10.1155/2013/917647 (2013). PMC3832963 24288570

[b5] Gonzales J. F. . Applying the precautionary principle to nutrition and cancer. J Am Coll Nutr. 33, 239–246 (2014). 24870117 10.1080/07315724.2013.866527

[b6] Mohamad H. . The effect of dietary and exercise interventions on body weight in prostate cancer patients: a systematic review. Nutr Cancer. 67, 43–60 (2015). 25425328 10.1080/01635581.2015.976313

[b7] Longo V. D. & Fontana L. Calorie restriction and cancer prevention: metabolic and molecular mechanisms. Trends Pharmacol Sci. 31, 89–98 (2010). 20097433 10.1016/j.tips.2009.11.004 PMC2829867

[b8] Yang C. S., Landau J. M., Huang M. T. & Newmark H. L. Inhibition of carcinogenesis by dietary polyphenolic compounds. Annu Rev Nutr. 21, 381–406 (2001). 11375442 10.1146/annurev.nutr.21.1.381

[b9] Glanz K. Behavioral research contributions and needs in cancer prevention and control: dietary change. Prev Med. 26, S43–S55 (1997). 9327492 10.1006/pmed.1997.0209

[b10] Rizza W., Veronese N. & Fontana L. What are the roles of calorie restriction and diet quality in promoting healthy longevity? Ageing Res Rev. 13, 38–45 (2014). 24291541 10.1016/j.arr.2013.11.002

[b11] Hietaniemi M. . The effect of a short-term hypocaloric diet on liver gene expression and metabolic risk factors in obese women. Nutr Metab Cardiovasc Dis. 19, 177–183 (2009). 18804985 10.1016/j.numecd.2008.06.009

[b12] Anson R. M. . Intermittent fasting dissociates beneficial effects of dietary restriction on glucose metabolism and neuronal resistance to injury from calorie intake. Proc Natl Acad Sci USA 100, 6216–6220 (2003). 12724520 10.1073/pnas.1035720100 PMC156352

[b13] Tapsell L. . Short term effects of energy restriction and dietary fat sub-type on weight loss and disease risk factors. Nutr Metab Cardiovasc Dis. 20, 317–325 (2010). 19570664 10.1016/j.numecd.2009.04.007

[b14] Silvera S. A. N., Jain M., Howe G. R., Miller A. B. & Rohan T. E. Energy balance and breast cancer risk: a prospective cohort study. Breast Cancer Res Treat. 97, 97–106 (2006). 16319973 10.1007/s10549-005-9098-3

[b15] Chang S. C. . Association of energy intake and energy balance with postmenopausal breast cancer in the prostate, lung, colorectal, and ovarian cancer screening trial. Cancer Epidemiol Biomarkers Prev. 15, 334–341 (2006). 16492925 10.1158/1055-9965.EPI-05-0479

[b16] Harvie M. & Howell A. Energy restriction and the prevention of breast cancer. Proc Nutr Soc. 71, 263–275 (2012). 22414375 10.1017/S0029665112000195

[b17] Heilbronn L. K., Smith S. R., Martin C. K., Anton S. D. & Ravussin E. Alternate-day fasting in nonobese subjects: effects on body weight, body composition, and energy metabolism. Am J Clin Nutr. 81, 69–73 (2005). 15640462 10.1093/ajcn/81.1.69

[b18] Halberg N. . Effect of intermittent fasting and refeeding on insulin action in healthy men. J Appl Physiol (1985). 99, 2128–2136 (2005). 16051710 10.1152/japplphysiol.00683.2005

[b19] Heilbronn L. K. . Glucose tolerance and skeletal muscle gene expression in response to alternate day fasting. Obes Res. 13, 574–581 (2005). 15833943 10.1038/oby.2005.61

[b20] Welsch C. W., House J. L., Herr B. L., Eliasberg S. J. & Welsch M. A. Enhancement of mammary carcinogenesis by high levels of dietary fat: a phenomenon dependent on ad libitum feeding. J Natl Cancer Inst. 82, 1615–1620 (1990). 2136369 10.1093/jnci/82.20.1615

[b21] Gillette C. A. . Energy availability and mammary carcinogenesis: effects of calorie restriction and exercise. Carcinogenesis. 18, 1183–1188 (1997). 9214601 10.1093/carcin/18.6.1183

[b22] Cleary M. P. . Prevention of mammary tumorigenesis by intermittent caloric restriction: does caloric intake during refeeding modulate the response? Exp Biol Med (Maywood). 232, 70–80 (2007). 17202587

[b23] Cleary M. P. . Weight-cycling decreases incidence and increases latency of mammary tumors to a greater extent than does chronic caloric restriction in mouse mammary tumor virus-transforming growth factor-alpha female mice. Cancer Epidemiol Biomarkers Prev. 11, 836–843 (2002). 12223427

[b24] Thompson H. J. . Effect of dietary energy restriction on vascular density during mammary carcinogenesis. Cancer Res. 64, 5643–5650 (2004). 15313902 10.1158/0008-5472.CAN-04-0787

[b25] Lv M., Zhu X., Wang H., Wang F. & Guan W. Roles of caloric restriction, ketogenic diet and intermittent fasting during initiation, progression and metastasis of cancer in animal models: a systematic review and meta-analysis. PloS one 9, e115147, 10.1371/journal.pone.0115147 (2014). 25502434 PMC4263749

[b26] Rogozina O. P., Bonorden M. J., Grande J. P. & Cleary M. P. Serum insulin-like growth factor-I and mammary tumor development in ad libitum-fed, chronic calorie-restricted, and intermittent calorie-restricted MMTV-TGF-alpha mice. Cancer Prev Res (Phila). 2, 712–719 (2009). 19654106 10.1158/1940-6207.CAPR-09-0028

[b27] Rogozina O. P., Nkhata K. J., Nagle E. J., Grande J. P. & Cleary M. P. The protective effect of intermittent calorie restriction on mammary tumorigenesis is not compromised by consumption of a high fat diet during refeeding. Breast Cancer Res Treat. 138, 395–406 (2013). 23446811 10.1007/s10549-013-2464-7 PMC3610797

[b28] Berrigan D., Perkins S. N., Haines D. C. & Hursting S. D. Adult-onset calorie restriction and fasting delay spontaneous tumorigenesis in p53-deficient mice. Carcinogenesis. 23, 817–822 (2002). 12016155 10.1093/carcin/23.5.817

[b29] Bonorden M. J. . Intermittent calorie restriction delays prostate tumor detection and increases survival time in TRAMP mice. Nutr Cancer. 61, 265–275 (2009). 19235043 10.1080/01635580802419798

[b30] Cleary M. P. & Grossmann M. E. The manner in which calories are restricted impacts mammary tumor cancer prevention. J Carcinog. 10, 21 (2011). 22013391 10.4103/1477-3163.85181 PMC3190408

[b31] Dogan S., Rogozina O. P., Lokshin A. E., Grande J. P. & Cleary M. P. Effects of chronic vs. intermittent calorie restriction on mammary tumor incidence and serum adiponectin and leptin levels in MMTV-TGF-alpha mice at different ages. Oncol Lett. 1, 167–176 (2010). 22966277 10.3892/ol_00000031 PMC3436387

[b32] Lanza-Jacoby S. . Calorie restriction delays the progression of lesions to pancreatic cancer in the LSL-KrasG12D; Pdx-1/Cre mouse model of pancreatic cancer. Exp Biol Med (Maywood). 238, 787–797 (2013). 23828595 10.1177/1535370213493727

[b33] Mizuno N. K. . Combination of intermittent calorie restriction and eicosapentaenoic acid for inhibition of mammary tumors. Cancer Prev Res (Phila). 6, 540–547 (2013). 23550153 10.1158/1940-6207.CAPR-13-0033 PMC4296517

[b34] Pape-Ansorge K. A., Grande J. P., Christensen T. A., Maihle N. J. & Cleary M. P. Effect of moderate caloric restriction and/or weight cycling on mammary tumor incidence and latency in MMTV-Neu female mice. Nutr Cancer. 44, 162–168 (2002). 12734063 10.1207/S15327914NC4402_07

[b35] Harris S. R., Brix A. E., Broderson J. R. & Bunce O. R. Chronic energy restriction versus energy cycling and mammary tumor promotion. Proc Soc Exp Biol Med. 209, 231–236 (1995). 7777584 10.3181/00379727-209-43897

[b36] Kritchevsky D., Welch C. B. & Klurfeld D. M. Response of mammary tumors to caloric restriction for different time periods during the promotion phase. Nutr Cancer. 12, 259–269 (1989). 2505241 10.1080/01635588909514025

[b37] Mehta R. S., Harris S. R., Gunnett C. A., Bunce O. R. & Hartle D. K. The effects of patterned calorie-restricted diets on mammary tumor incidence and plasma endothelin levels in DMBA-treated rats. Carcinogenesis. 14, 1693–1696 (1993). 8353853 10.1093/carcin/14.8.1693

[b38] Tagliaferro A. R. . Cyclic food restriction alters substrate utilization and abolishes protection from mammary carcinogenesis female rats. J Nutr. 126, 1398–1405 (1996). 8618136 10.1093/jn/126.5.1398

[b39] Zhu Z., Jiang W., McGinley J., Wolfe P. & Thompson H. J. Effects of dietary energy repletion and IGF-1 infusion on the inhibition of mammary carcinogenesis by dietary energy restriction. Mol Carcinog. 42, 170–176 (2005). 15599926 10.1002/mc.20071

[b40] Hooijmans C. R. . SYRCLE’s risk of bias tool for animal studies. BMC Med Res Methodol. 14, 43 (2014). 24667063 10.1186/1471-2288-14-43 PMC4230647

[b41] Compalati E. . Systematic review on the efficacy of fexofenadine in seasonal allergic rhinitis: a meta-analysis of randomized, double-blind, placebo-controlled clinical trials. Int Arch Allergy Immunol. 156, 1–15 (2011). 21969990 10.1159/000321896

[b42] Zhou J. G. . Treatment on advanced NSCLC: platinum-based chemotherapy plus erlotinib or platinum-based chemotherapy alone? A systematic review and meta-analysis of randomised controlled trials. Med Oncol. 32, 471 (2015). 25579169 10.1007/s12032-014-0471-0

[b43] Fleiss J. L., Levin B. & Paik M. C. Statistical Methods for Rates and Proportions. 3rd ed. (New York:Wiley, 2003).

[b44] DerSimonian R. & Laird N. Meta-analysis in clinical trials. Control Clin Trials. 7, 177–188 (1986). 3802833 10.1016/0197-2456(86)90046-2

[b45] Higgins J. P. T., Thompson S. G., Deeks J. J. & Altman D. G. Measuring inconsistency in meta-analyses. BMJ. 327, 557–560 (2003). 12958120 10.1136/bmj.327.7414.557 PMC192859

[b46] Duval S. & Tweedie R. A Nonparametric “Trim and Fill” Method of Accounting for Publication Bias in Meta-Analysis. J Am Stat Assoc. 95, 89–98 (2000).

[b47] Klurfeld D. M., Welch C. B., Davis M. J. & Kritchevsky D. Determination of degree of energy restriction necessary to reduce DMBA-induced mammary tumorigenesis in rats during the promotion phase. J Nutr. 119, 286–291 (1989). 2493082 10.1093/jn/119.2.286

[b48] Birt D. F., Pelling J. C., White L. T., Dimitroff K. & Barnett T. Influence of diet and calorie restriction on the initiation and promotion of skin carcinogenesis in the SENCAR mouse model. Cancer Res. 51, 1851–1854 (1991). 1900738

[b49] Sugie S., Tanaka T., Mori H. & Reddy B. S. Effect of restricted caloric intake on the development of the azoxymethane-induced glutathione S-transferase placental form positive hepatocellular foci in male F344 rats. Cancer Lett. 68, 67–73 (1993). 8422651 10.1016/0304-3835(93)90221-t

[b50] Dirx M. J., Zeegers M. P., Dagnelie P. C., van den Bogaard T. & van den Brandt P. A. Energy restriction and the risk of spontaneous mammary tumors in mice: a meta-analysis. Int J Cancer. 106, 766–770 (2003). 12866038 10.1002/ijc.11277

[b51] Gross L. & Dreyfuss Y. Reduction in the incidence of radiation-induced tumors in rats after restriction of food intake. Proc Natl Acad Sci USA 81, 7596–7598 (1984). 6594701 10.1073/pnas.81.23.7596 PMC392194

[b52] Thompson H. J. & McTiernan A. Weight cycling and cancer: weighing the evidence of intermittent caloric restriction and cancer risk. Cancer Prev Res (Phila). 4, 1736–1742 (2011). 21982873 10.1158/1940-6207.CAPR-11-0133 PMC3208747

[b53] Martin-Montalvo A., Villalba J. M., Navas P. & de Cabo R. NRF2, cancer and calorie restriction. Oncogene. 30, 505–520 (2011). 21057541 10.1038/onc.2010.492 PMC4684645

[b54] Bordone L. & Guarente L. Calorie restriction, SIRT1 and metabolism: understanding longevity. Nat Rev Mol Cell Biol. 6, 298–305 (2005). 15768047 10.1038/nrm1616

[b55] Simone B. A. . Selectively starving cancer cells through dietary manipulation: methods and clinical implications. Future oncology. 9, 959–976 (2013). 23837760 10.2217/fon.13.31

[b56] Klement R. J. & Kammerer U. Is there a role for carbohydrate restriction in the treatment and prevention of cancer? Nutrition & metabolism 8, 75, 10.1186/1743-7075-8-75 (2011). 22029671 PMC3267662

[b57] Keenan M. M. & Chi J. T. Alternative fuels for cancer cells. Cancer J. 21, 49–55 (2015). 25815843 10.1097/PPO.0000000000000104 PMC4380238

[b58] Ghaffari P., Mardinoglu A. & Nielsen J. Cancer Metabolism: A Modeling Perspective. Front Physiol. 6, 382 (2015). 26733270 10.3389/fphys.2015.00382 PMC4679931

[b59] Varady K. A. & Hellerstein M. K. Alternate-day fasting and chronic disease prevention: a review of human and animal trials. Am J Clin Nutr. 86, 7–13 (2007). 17616757 10.1093/ajcn/86.1.7

[b60] Bonorden M. J. . Cross-sectional analysis of intermittent versus chronic caloric restriction in the TRAMP mouse. Prostate. 69, 317–326 (2009). 19016490 10.1002/pros.20878

[b61] Klement R. J. & Champ C. E. Calories, carbohydrates, and cancer therapy with radiation: exploiting the five R’s through dietary manipulation. Cancer Metastasis Rev. 33, 217–229 (2014). 24436017 10.1007/s10555-014-9495-3 PMC3988521

[b62] Saleh A. D. . Caloric restriction augments radiation efficacy in breast cancer. Cell cycle. 12, 1955–196 (2013). 23708519 10.4161/cc.25016 PMC3735710

[b63] Rogozina O. P., Bonorden M. J., Seppanen C. N., Grande J. P. & Cleary M. P. Effect of chronic and intermittent calorie restriction on serum adiponectin and leptin and mammary tumorigenesis. Cancer Prev Res (Phila). 4, 568–581 (2011). 21257708 10.1158/1940-6207.CAPR-10-0140 PMC3071428

[b64] Jiang N., Sun R. & Sun Q. Leptin signaling molecular actions and drug target in hepatocellular carcinoma. Drug Des Devel Ther. 8, 2295–2302 (2014). 10.2147/DDDT.S69004 PMC4238752 25484575

[b65] Ray A., Nkhata K. J. & Cleary M. P. Effects of leptin on human breast cancer cell lines in relationship to estrogen receptor and HER2 status. Int J Oncol. 30, 1499–1509 (2007). 17487372

[b66] Garofalo C., Sisci D. & Surmacz E. Leptin interferes with the effects of the antiestrogen ICI 182,780 in MCF-7 breast cancer cells. Clin Cancer Res. 10, 6466–6475 (2004). 15475434 10.1158/1078-0432.CCR-04-0203

[b67] Grossmann M. E., Nkhata K. J., Mizuno N. K., Ray A. & Cleary M. P. Effects of adiponectin on breast cancer cell growth and signaling. Br J Cancer. 98, 370–379 (2008). 18182989 10.1038/sj.bjc.6604166 PMC2361440

[b68] Takahata C. . Demonstration of adiponectin receptors 1 and 2 mRNA expression in human breast cancer cells. Cancer Lett. 250, 229–236 (2007). 17123704 10.1016/j.canlet.2006.10.006

[b69] Grossmann M. E. & Cleary M. P. The Balance between Leptin and Adiponectin in the Control of Carcinogenesis- Focus on Mammary Tumorigenesis. Biochimie. 94, 2164–2171 (2012). 22728769 10.1016/j.biochi.2012.06.013 PMC4296518

[b70] O’Neill I. K. & Fishbein L. An IARC Manual series aimed at assisting cancer epidemiology and prevention. “Environmental carcinogens: selected methods of analysis”. Int J Environ Anal Chem. 26, 229–240 (1986). 3771058 10.1080/03067318608077117

[b71] Pratt M. M. . Polycyclic aromatic hydrocarbon (PAH) exposure and DNA adduct semi-quantitation in archived human tissues. Int J Environ Res Public Health. 8, 2675–2691 (2011). 21845152 10.3390/ijerph8072675 PMC3155323

[b72] Alshafie G. A. . Inhibition of mammary tumor growth by a novel nontoxic retinoid: chemotherapeutic evaluation of a C-linked analog of 4-HPR-glucuronide. Anticancer Res. 25, 2391–2398 (2005). 16082771

[b73] Rao A. R. . Effective inhibition of skin cancer, tyrosinase, and antioxidative properties by astaxanthin and astaxanthin esters from the green alga Haematococcus pluvialis. J Agric Food Chem. 61, 3842–3851 (2013). 23473626 10.1021/jf304609j

[b74] Iino Y. . Effect of medroxyprogesterone acetate (MAP) and vitamin C on DMBA induced rat mammary cancer in relation to estrogen receptor (ER). Gan No Rinsho. 29, 949–954 (1983). 6225884

[b75] Bikle D. D. . Protective role of vitamin D signaling in skin cancer formation. J Steroid Biochem Mol Biol. 136, 271–279 (2013). 23059470 10.1016/j.jsbmb.2012.09.021 PMC3596439

[b76] Zinser G. M., Suckow M. & Welsh J. Vitamin D receptor (VDR) ablation alters carcinogen-induced tumorigenesis in mammary gland, epidermis and lymphoid tissues. J Steroid Biochem Mol Biol. 97, 153–164 (2005). 16111884 10.1016/j.jsbmb.2005.06.024

[b77] Dhanarasu S., Selvam M., Salama S. M., Shanmugam M. & Sethuraman P. Terminalia Arjuna (Roxb.) Modulates Circulatory Antioxidants on 7,12-dimethylbenz(a)anthracene- induced Hamster Buccal Pouch Carcinogenesis. Oman Med J. 25, 276–281 (2010). 22043357 10.5001/omj.2010.81 PMC3191666

[b78] Krishnaveni M. & Mirunalini S. Chemopreventive efficacy of Phyllanthus emblica L. (amla) fruit extract on 7,12-dimethylbenz(a)anthracene induced oral carcinogenesis–a dose-response study. Environ Toxicol Pharmacol. 34, 801–810 (2012). 23058484 10.1016/j.etap.2012.09.006

[b79] Hursting S. D., Lavigne J. A., Berrigan D., Perkins S. N. & Barrett J. C. Calorie restriction, aging, and cancer prevention: Mechanisms of action and a applicability to humans. Annu Rev Med. 54, 131–152 (2003). 12525670 10.1146/annurev.med.54.101601.152156

[b80] Chen Q. . Pharmacologic doses of ascorbate act as a prooxidant and decrease growth of aggressive tumor xenografts in mice. Proc Natl Acad Sci USA 105, 11105–11109 (2008). 18678913 10.1073/pnas.0804226105 PMC2516281

