# Supplementary material for: Effect of Intermittent versus Chronic Calorie Restriction on Tumor Incidence: A Systematic Review and Meta-Analysis of Animal Studies
Source: Sci Rep. 2016 Sep 22;6:33739. doi: 10.1038/srep33739 (PMC5031958; doi:10.1038/srep33739)
Supplement: Supplementary Information [file srep33739-s1.pdf]

Supplementary Information for:

Effect of Intermittent versus Chronic Calorie Restriction on Tumor Incidence: A Systematic Review and Meta-Analysis of Animal Studies

Running title: Intermittent *vs*. chronic calorie restriction on tumor incidence

Yalan Chen, MS[1,2,*], Lifeng Ling, MS[1,3,*], Guanglei Su, BsC[1], Ming Han, BsC[1], Xikang Fan, BsC[1], PengchengXun, MD, PhD[4,†], and Guangfei Xu, MD, PhD[1,†]

[1]Department of Nutrition and Food Science, School of Public Health, Nantong University, Nantong, Jiangsu, China;

[2]Department of Medical Informatics, School of Medicine, Nantong University, Nantong, Jiangsu, China;

[3]Department of Human Resources, Nantong University, Nantong, Jiangsu, China;

[4]Department of Epidemiology and Biostatistics, School of Public Health-Bloomington, Indiana University, Bloomington, IN, USA.

*These authors contributed equally to this work.

[†]**Corresponding authors:**

Dr. Guangfei Xu; Department of Nutrition and Food Science, School of Public Health, Nantong University, No 9 Seyuan Rd, Nantong, Jiangsu 226019, China; Phone: 86-0513-8501-2911; E-mail: xugf@ntu.edu.cn

Dr. Pengcheng Xun; Department of Epidemiology and Biostatistics, School of Public Health-Bloomington, Indiana University, 1025 E. Seventh Street, C103, Bloomington, Indiana 47405, USA; Phone: 01-812-856-0772; Fax: 01-812-855-9907; E-mail: pxun@indiana.edu

Supplementary Table 1. Assessment of Study Quality and Risk of Bias According to SYRCLE's RoB Tool.

Study	# 1	# 2	# 3	# 4	# 5	# 6	# 7	# 8	# 9	# 10	Quality
**Genetically engineered mouse models**											
Berrigan, 2002	Yes	Yes	No	Yes	No	Unclear	Yes	No	Yes	Yes	6
Cleary, 2002	Unclear	Yes	No	Yes	No	Unclear	Yes	Yes	Yes	Yes	6
Pape-Ansorge, 2002	Unclear	Yes	No	Yes	No	Unclear	Yes	Yes	Yes	Yes	6
Cleary, 2007	Unclear	Yes	No	Yes	No	Unclear	Yes	Yes	Yes	Yes	6
Bonorden, 2009	Yes	Yes	No	Yes	No	Unclear	Yes	Yes	Yes	Yes	7
Rogozina, 2009	Unclear	Yes	No	Yes	No	Unclear	Yes	Yes	No	Yes	5
Dogan, 2010	Yes	Yes	No	Yes	No	Unclear	Yes	Yes	No	Yes	6
Lanza-Jacoby, 2013	Yes	Yes	No	Yes	No	Unclear	Yes	No	Yes	Yes	6
Mizuno, 2013	Unclear	Yes	No	Yes	No	Unclear	Yes	No	Yes	Yes	5
Rogozina, 2013	Unclear	Yes	No	Yes	No	Unclear	Yes	No	Yes	Yes	5
Grossmann, unpublished	Unclear	Unclear	No	Unclear	Unclear	Unclear	Unclear	Unclear	Unclear	Unclear	0
**Chemically induced rat models**											
Kritchevsky, 1989	Yes	Yes	No	Yes	No	Unclear	Yes	Yes	Yes	Yes	7
Mehta, 1993	Yes	Yes	No	Yes	No	Unclear	Yes	Yes	Yes	Yes	7
Harris, 1995	Yes	Yes	No	Yes	No	Unclear	Yes	Yes	Yes	Yes	7
Tagliaferro, 1996	Yes	Yes	No	Yes	No	Unclear	Yes	No	Yes	Yes	6
Zhu, 2005	Unclear	Yes	No	Yes	No	Unclear	Yes	Yes	Yes	Yes	6

SYRCLE's RoB: SYstematic Review Centre for Laboratory animal Experimentation; Item 1: sequence generation; 2: baseline characteristics; 3: allocation concealment; 4: random housing; 5: performance blinding; 6: random outcome assessment; 7: detection blinding; 8: incomplete outcome data adequately addressed; 9: free of selective outcome reporting; 10: free of other sources of bias; Yes: low risk of bias; no: high risk of bias; unclear: unclear risk of bias. Higher quality score represents lower the risk bias.

Supplementary Table 2. PRISMA 2009 Checklist.

Section/topic	#	Checklist item	Reported on page #
**TITLE**			
Title	1	Identify the report as a systematic review, meta-analysis, or both.	1
**ABSTRACT**			
Structured summary	2	Provide a structured summary including, as applicable: background; objectives; data sources; study eligibility criteria, participants, and interventions; study appraisal and synthesis methods; results; limitations; conclusions and implications of key findings; systematic review registration number.	2
**INTRODUCTION**			
Rationale	3	Describe the rationale for the review in the context of what is already known.	3
Objectives	4	Provide an explicit statement of questions being addressed with reference to participants, interventions, comparisons, outcomes, and study design (PICOS).	4
**METHODS**			
Protocol and registration	5	Indicate if a review protocol exists, if and where it can be accessed (e.g., Web address), and, if available, provide registration information including registration number.	
Eligibility criteria	6	Specify study characteristics (e.g., PICOS, length of follow-up) and report characteristics (e.g., years considered, language, publication status) used as criteria for eligibility, giving rationale.	5
Information sources	7	Describe all information sources (e.g., databases with dates of coverage, contact with study authors to identify additional studies) in the search and date last searched.	4
Search	8	Present full electronic search strategy for at least one database, including any limits used, such that it could be repeated.	4
Study selection	9	State the process for selecting studies (i.e., screening, eligibility, included in systematic review, and, if applicable, included in the meta-analysis).	5
Data collection process	10	Describe method of data extraction from reports (e.g., piloted forms, independently, in duplicate) and any processes for obtaining and confirming data from investigators.	6
Data items	11	List and define all variables for which data were sought (e.g., PICOS, funding sources) and any assumptions and simplifications made.	6
Risk of bias in individual studies	12	Describe methods used for assessing risk of bias of individual studies (including specification of whether this was done at the study or outcome level), and how this information is to be used in any data synthesis.	7
Summary measures	13	State the principal summary measures (e.g., risk ratio, difference in means).	7
Synthesis of results	14	Describe the methods of handling data and combining results of studies, if done, including measures of consistency (e.g., I^2) for each meta-analysis.	7
Risk of bias across studies	15	Specify any assessment of risk of bias that may affect the cumulative evidence (e.g., publication bias, selective reporting within studies).	7
Additional analyses	16	Describe methods of additional analyses (e.g., sensitivity or subgroup analyses, meta-regression), if done, indicating which were pre-specified.	8
**RESULTS**			
Study selection	17	Give numbers of studies screened, assessed for eligibility, and included in the review, with reasons for exclusions at each stage, ideally with a flow diagram.	8-9
Study characteristics	18	For each study, present characteristics for which data were extracted (e.g., study size, PICOS, follow-up period) and provide	9-10

		the citations.	
Risk of bias within studies	19	Present data on risk of bias of each study and, if available, any outcome level assessment (see item 12).	10-12
Results of individual studies	20	For all outcomes considered (benefits or harms), present, for each study: (a) simple summary data for each intervention group (b) effect estimates and confidence intervals, ideally with a forest plot.	9-10
Synthesis of results	21	Present results of each meta-analysis done, including confidence intervals and measures of consistency.	10-12
Risk of bias across studies	22	Present results of any assessment of risk of bias across studies (see Item 15).	10-12
Additional analysis	23	Give results of additional analyses, if done (e.g., sensitivity or subgroup analyses, meta-regression [see Item 16]).	12
**DISCUSSION**			
Summary of evidence	24	Summarize the main findings including the strength of evidence for each main outcome; consider their relevance to key groups (e.g., healthcare providers, users, and policy makers).	12
Limitations	25	Discuss limitations at study and outcome level (e.g., risk of bias), and at review-level (e.g., incomplete retrieval of identified research, reporting bias).	16
Conclusions	26	Provide a general interpretation of the results in the context of other evidence, and implications for future research.	19
**FUNDING**			
Funding	27	Describe sources of funding for the systematic review and other support (e.g., supply of data); role of funders for the systematic review.	20

Supplementary Table 3. Baseline Characteristics of the Studies Included in This Meta-analysis.

Study	Group	Animal type	Tumor type	Feeding regimens	Trial length, W	Body weight, g
**Genetically engineered mouse models**						
Berrigan, 2002	ICR	p53+/- M	Multiple	1 day/W of fasting 6 days/W of AL (AIN-76A)	48	38
	CCR	p53+/- M	Multiple	60% AL (AIN-76A)	48	27
Cleary, 2002	ICR	TGF-a/Lep$^+$ Lep^{ab} FM	Mammary	3 weeks of 50% AL (AIN-93-mod)<br>3 weeks of 100% AL (AIN-93M)	72	27.2±1.1
	CCR	TGF-a/Lep$^+$Lep^{ab} FM	Mammary	2:1 of AIN-93M:AIN-93-mod	72	26.9± 0.9
Pape-Ansorge, 2002	ICR	MMTVNeu FM	Mammary	3 weeks of 50% AL (AIN-93-mod)<br>3 weeks of 100% AL (AIN-93M)	73	31.1 ± 0.8
	CCR	MMTVNeu FM	Mammary	2:1 of AIN-93M:AIN-93-mod	73	28.0 ± 0.8
Cleary, 2007	ICR	MMTV-TGF-a/Lepr$^+$Lepr^{db} FM	Mammary	3 weeks of 50% AL (AIN-93-mod)<br>3 weeks of 100% AL (AIN-93M)	76	R:25±0.7<br>RF:32.5±0.6
	CCR	MMTV-TGF-a/Lepr$^+$Lepr^{db} FM	Mammary	2:1 of AIN-93M:AIN-93-mod	76	26.2±0.5
Bonorden, 2009	ICR	TRAMP M	Prostate	2 weeks of 50% AL (AIN-93M)<br>2 weeks of 100% AL (AIN-93M)	49	R:27.4±0.7<br>RF:30.9±0.6
	CCR	TRAMP M	Prostate	75% AL (AIN-93M)	50	29.2 ± 0.4
Rogozina, 2009	ICR	MMTV-TGF-α FM	Mammary	3 weeks of 50% AL (AIN-93-mod)<br>3 weeks of 100% AL (AIN-93M)	74	R:24.3±0.5<br>RF:27.4±0.5
	CCR	MMTV-TGF-α FM	Mammary	75% AL (AIN-93M)	74	27.2±0.4
Dogan, 2010	ICR	MMTV-TGF-a FM	Mammary	3 weeks of 50% AL (AIN-93-mod)<br>3 weeks of 100% AL (AIN-93M)	74	R:22.6±0.4<br>RF:26.7±0.6
	CCR	MMTV-TGF-a FM	Mammary	75% AL (AIN-93M)	74	25.1±0.6
Lanza-Jacoby, 2013	ICR	LSL-Kras^{G12D};Pdx-1/Cre M	Pancreatic	3 weeks of 50% AL (AIN-93-mod)<br>3 weeks of 100% AL (AIN-93M)	38	21.7 ±0.4
	CCR	LSL-Kras^{G12D};Pdx-1/Cre M	Pancreatic	2:1 of AIN-93M:AIN-93-mod	38	21.0 ±0.5
Mizuno, 2013	ICR	MMTV-Her2/neu M	Mammary	3 weeks of 50% AL (AIN-93M)<br>3 weeks of 100% AL (AIN-93M)	52	NA
	CCR	MMTV-Her2/neu M	Mammary	75% AL (AIN-93M)	52	NA
Rogozina, 2013	ICR	MMTV-TGF-α FM	Mammary	3 weeks of 50% AL (19% fat by calories)<br>3 weeks of 100% AL (33.6% fat by calories)	74	R:23.3±0.7*<br>RF:29.3±0.7*
	CCR	MMTV-TGF-α FM	Mammary	75% AL (29.6% fat by calories)	74	28.9±0.71*
Grossmann, unpublished	ICR	TGF-a/Lep+ Lepab FM	Mammary	3 weeks of 50% AL (AIN-93-mod)<br>3 weeks of 100% AL (AIN-93M)	72	NA
	CCR	TGF-a/Lep+ Lepab FM	Mammary	2:1 of AIN-93M:AIN-93-mod	72	NA
**Chemically induced rat models**						

Kritchevsky, 1989	ICR	Sprague-Dawley FM	Mammary	1.75 months of 25% AL<br>2.25 months of 100% AL	16	325.8
	CCR	Sprague-Dawley FM	Mammary	75% AL	16	276
Mehta, 1993	ICR	Sprague-Dawley FM	Mammary	2 days of 60% AL<br>2days of 100% AL (AIN-76)	10	275±4.4
	CCR	Sprague-Dawley FM	Mammary	60% AL (AIN-76)	10	234±2.8
Harris, 1995	ICR	Sprague-Dawley FM	Mammary	2days of 60% AL<br>2 days of 100%AL (AIN-76)	10	274.85*
	CCR	Sprague-Dawley FM	Mammary	60% AL (AIN-76)	10	233.92*
Tagliaferro, 1996	ICR	Sprague-Dawley FM	Mammary	1 week of 67% AL<br>3 weeks of 100% AL	18	293.1±2.2
	CCR	Sprague-Dawley FM	Mammary	60% AL	18	299.7±3.9
Zhu, 2005	ICR	Sprague-Dawley FM	Multiple	6 weeks of 60% AL<br>8days of 100% AL (AIN-76)	7	160±2.0
	CCR	Sprague-Dawley FM	Multiple	60% AL	7	139±1.0

*Data were extracted from the original figures using GetData Graph Digitizer.
Values are means ± SE.

AIN-93-mod: 2-fold increase in protein, fat, vitamins, and minerals, and fat contents and was formulated to be isocaloric with AIN-93M diet; AL: ad libitum feeding; CCR: chronic calorie restriction; FM: female mice; ICR: intermittent calorie restriction; M: mice; MMTV: mouse mammary tumour virus; NA: not available; R: results of ICR restriction periods; RF: results of ICR refeeding periods; SE: standard error; TGF: transforming growth factor; W: week.

Supplementary Table 4. Influence of Statiticcal Model Selection on Summary Estimate: A Sensitivity Analysis.

Indexes	Animal models	Fixed-effects model	Random-effects model
		**RR/SMD (95% CI)**	**RR/SMD (95% CI)**
**Tumor incidence**	Genetically engineered	0.69 (0.61, 0.78)	0.57 (0.37, 0.88)
	Chemically induced	1.53 (1.25, 1.87)	1.53 (1.13, 2.06)
**IGF-1**	Genetically engineered	-0.72 (-0.98, -0.46)	-0.74 (-1.17, -0.31)
**Leptin**	Genetically engineered	-0.64 (-0.98, -0.29)	-0.64 (-0.98, -0.29)
**Adiponectin**	Genetically engineered	**0.65 (0.32, 0.97)**	**0.68 (-0.02, 1.38)**
**No. of tumors/mouse**	Genetically engineered	-0.51 (-1.14,0.12)	-0.51 (-1.14, 0.12)
	Chemically induced	0.30 (-0.09, 0.70)	0.63 (-1.31, 2.57)
**Age at detection**	Genetically engineered	0.17 (-0.38, 0.73)	0.17 (-0.38, 0.73)
**Tumor weight**	Genetically engineered	0.15 (-0.41, 0.71)	0.15 (-0.65, 0.94)

CI: confidence interval; IGF-1: insulin-like growth factor 1; SMD: standardised mean differences; RR: relative risk (for tumor incidence).

Supplementary Table 5. Influence of Single Studies on Summary Estimate Using Random-effects Model: A Sensitivity Analysis.

Outcome	Animal models	Study omitted	RR/SMD (95% CI)
**Tumor incidence**	**Genetically engineered mouse models**	None	**0.57(0.36, 0.88)**
		Berrigan,2002	0.48 (0.25, 0.94)
		Cleary,2002	0.63 (0.42, 0.94)
		Pape-Ansorge,2002	0.55 (0.35, 0.89)
		Cleary,2006	0.59 (0.38, 0.92)
		Bonorden,2009	0.52 (0.31, 0.85)
		Rogozina,2009	0.64 (0.43, 0.96)
		Dogan,2010	0.57 (0.36, 0.90)
		Lanza-Jacoby,2013	0.56 (0.35, 0.89)
		Mizuno,2013	0.49 (0.29, 0.84)
		Rogozina,2013	0.67 (0.47, 0.96)
		Grossmann, unpublished	0.54(0.33, 0.88)
	**Chemically induced rat models**	None	**1.53(1.13, 2.06)**
		Kritchevsky,1989	1.46(1.09, 1.97)
		Mehta RS,1993	1.43(1.06, 1.92)
		Harris,1995	1.37(1.06, 1.76)
		Tagliaferro,1996	1.84(1.08, 3.16)
		Zhu,2005	1.74(1.11, 2.73)
**IGF-1**	**Genetically engineered mouse models**	None	**-0.74(-1.17, -0.31)**
		Cleary,2006	-0.87(-1.31, -0.04)
		Bonorden,2009	-0.66(-1.19, -0.14)
		Rogozina,2009	-0.73(-1.31, -0.16)
		Dogan,2010	-0.61(-1.00, -0.23)
		Rogozina,2013	-0.85(-1.35, -0.35)
**Leptin**	**Genetically engineered mouse models**	None	**-0.64(-0.98, -0.29)**
		Bonorden,2009	-0.53(-1.15, 0.09)
		Dogan,2010	-0.60(-0.97, -0.23)
		Rogozina,2013	-0.71(-1.08, -0.34)
**Adiponectin**	**Genetically engineered mouse models**	None	**0.68(-0.02, 1.38)**
		Bonorden,2009	0.68(-0.74, 2.11)
		Dogan,2010	0.44(-0.33, 1.21)
		Rogozina,2013	0.96(0.39, 1.53)

CI: confidence interval; IGF-1: insulin-like growth factor 1; SMD: standardised mean differences; RR: relative risk (for tumor incidence).
